# Gut microbiota and cognitive development in infant mice: Quantity and source of potable water

**DOI:** 10.1371/journal.pone.0286951

**Published:** 2023-06-14

**Authors:** Chong-Su Kim, Dong-Mi Shin

**Affiliations:** 1 Department of Food and Nutrition, College of Human Ecology, Seoul National University, Seoul, Republic of Korea; 2 Research Institute of Human Ecology, Seoul National University, Seoul, Republic of Korea; University of Jeddah, SAUDI ARABIA

## Abstract

Not only the water quantity consumed but also the source of drinking water has been considered for their health benefits, but there is limited evidence. We aimed to determine whether the amount and type of drinking water affect physiological and biological functions, including brain function, by confirming how it affects gut microbiota which has an important regulatory role in host physiology. Three-week-old infant mice were subjected to 1) a water restriction experiment (control group, *ad libitum* consumption of distilled water; dehydration group, time-limited access to distilled water [15 min/day]) and 2) different water source experiment (distilled water, purified water, spring water, and tap water groups). The gut microbiota and cognitive development were analyzed using the 16S ribosomal ribonucleic acid sequencing method and the Barnes maze, respectively. The relative abundance of Firmicutes and Bacteroidetes and the Firmicutes-to-Bacteroidetes ratio (F/B ratio) changed depending on age (juveniles vs. infants). Insufficient water intake reversed these developmental changes, showing that the relative abundances of Bacteroidetes and Firmicutes and the F/B ratio in dehydrated juvenile mice were similar to those in normal infant mice. Additionally, clustering analysis revealed no significant differences in the intestinal flora in the mice from the different drinking water sources; however, dehydration significantly altered the composition of the genera compared to the other water source groups wherein water was provided *ad libitum*. Moreover, cognitive development was significantly disrupted by insufficient water intake, although the type of drinking water had no significant influence. Cognitive decline, measured by relative latency, was positively associated with the relative abundance of unclassified Erysipelotrichaceae that were in significantly high relative abundance in the dehydration group. These results suggest that the water quantity consumed, rather than the mineral content of drinking water, is imperative for shaping the early gut microbiota associated with cognitive development during infancy.

## Introduction

Adequate water intake is essential for the body’s normal biological and physiological functions [1, 2]. Insufficient drinking water consumption during the developmental period is related to various health outcomes, including altered hippocampal gene expression related to cognitive development, abnormal physical growth, and renal development [3, 4]. Moreover, not only the water quantity consumed but also the source of drinking water has been considered for their health benefits. Water provides various minerals, including both macro-elements (e.g., calcium, magnesium, bicarbonate, and sulfate) and micro-elements (e.g., molybdenum and selenium), which may differ with various sources of drinking water [5]. Therefore, the demand for bottled mineral water has increased [6], although most people in the United States and Asia have access to clean and safe tap water [7].

Currently, the toxicity levels of minerals concerning human health are well-defined by the World Health Organization, European Union, and US Environmental Protection Agency guidelines, but the health benefits of minerals in drinking water are not fully understood [5]. In fact, over the past decades, whether the mineral constituents of drinking water have health-promoting effects in humans has been debated [8, 9]. Many studies have reported that highly mineralized water found in natural underground and mineral springs may have health benefits. A randomized controlled trial showed that drinking natural mineral water rich in magnesium and sodium sulfate for 6 weeks significantly improved stool consistency and health-related quality of life in individuals with functional constipation compared to placebo water with low mineral content [10]. However, there is limited evidence regarding the differences in health benefits of drinking water from various sources with different mineral compositions, including tap water, bottled water, and purified water, which are routinely consumed.

The gut microbiota is a complex ecosystem of microorganisms in the human gut that plays important roles in human health by affecting various biological and physiological functions [11]. Variation in the intestinal microfloral community depends on various factors, such as race, living environment, diet, disease presence, and age [11]. The early microbiota during infancy progresses toward a more complex structure similar to that of adults [12, 13]. In addition, normal development of the gut microbiota is important because it is a health-determining factor in adulthood [12–14]. During the evolution of the gut microbiota through dynamic changes toward a mature form, diet is an important factor in determining microbial communities [11–13]. Considering that drinking water is a dietary component and large amounts are consumed daily, its intake and source may be the key factors affecting the gut microbiota and overall human health.

The gut-brain axis involves communication between the gut microbiota and the brain [15, 16]. Several studies have demonstrated that signals from intestinal bacteria are critical for normal brain function throughout an individual’s lifecycle [16, 17]. In particular, alterations in the gut microbiota during the developmental period have been discussed for their regulatory roles in brain development and behavior during early life [16, 18]. Therefore, the potential association between developing microbiota and brain development has been largely discussed. Studies in germ-free young rodents without intestinal bacteria have shown impairments in object recognition memory and working memory in adulthood [19–21]. In addition, recovered gut microbial communities by dietary intervention during the developmental period reversed behavioral abnormalities such as persistent fear memories in infant rats [22].

In a previous study, our group confirmed that drinking water intake as part of the diet had an important influence on cognitive development by showing that dehydration during infancy resulted in abnormal cognitive development in infant mice [3]. The present study aimed to determine whether changes in the gut microbiota were associated with cognitive decline in dehydrated mice through the gut-brain axis. The effects of different drinking water sources on gut microbial composition during infancy have not yet been investigated. Therefore, the present study aimed to explore the effects of different mineral contents of water on the fecal microbial community in association with the gut-brain axis.

## Materials and methods

### Animals

Three-week-old male C57BL/6 mice were maintained under conventional conditions with a 12-h light/dark cycle and fed *ad libitum* with an AIN-93G diet. Animals in the same group were co-housed throughout the course of the experiments. The following were recorded every day, both before and after providing access to water: body weight, food intake, and water intake. All efforts were made to minimize the animal’s suffering during experimental procedures using CO_2_ overdose. Mice were sacrificed after 12 h of fasting by intraperitoneal injection of urethane. After the mice were sacrificed, blood and fecal samples were collected. All experimental procedures were approved by the Institutional Animal Care and Use Committee of Seoul National University (Approval number: SNU-140421-3) and conducted in accordance with its prescribed guidelines. The study was carried out in compliance with the ARRIVE guidelines.

### Experimental design

The experimental study design is shown in **S1 Fig**. Three-week-old mice were subjected to the 1) water restriction experiment or 2) different water source experiment. Except for the mice in the infant groups that were sacrificed at the age of 4 weeks, the rest of the groups were housed for 4 weeks and sacrificed at the age of 7 weeks, corresponding to juvenile age.

#### Experiment 1—Water restriction experiment

Animals were randomly assigned to one of the following experimental groups: control groups (infant mice group, n = 5; juvenile mice group, n = 6); dehydration groups (infant mice group, n = 6; juvenile mice group, n = 8). Animals from the dehydration groups were mildly dehydrated according to the daily water restriction protocol, as previously described [3]. Briefly, mice in the water restriction groups had limited access to water bottles (15 min/d) to induce mild dehydration, whereas animals in the control groups consumed water *ad libitum*. Both the dehydration and the control groups were provided with distilled water. Whether dehydration was induced in animals was assessed by evaluating the plasma osmolality level and brain vasopressin expression level as described in a previous study [3].

#### Experiment 2—Different water source experiment

Animals were randomly assigned to the following experimental groups: distilled water (DSW; n = 6), purified water (PUR; n = 6), spring water (SPR; n = 6), and tap water group (TAP; n = 6). Animals in the DSW, PUR, SPR, and TAP groups consumed water *ad libitum*. Purified water was provided from a Coway filtration appliance (Coway Co., Seoul, Republic of Korea), and bottled Evian spring water (Evian, Evian-les-Bains, France) was used for the spring water group. The water components from the different water sources were assessed by a water sample provider (Coway Co., Seoul, Republic of Korea) (**S1 Table**).

### Serum biochemical analysis

Serum was isolated from the blood by collecting blood samples in serum-separating tubes. A centrifugation at 3000 rpm and 4°C for 20 min yielded serum immediately stored at −80°C for later analysis. Serum biochemical indices, including serum glucose (mg/dL), total cholesterol (g/dL), blood urea nitrogen (mg/dL), total bilirubin (mg/dL), glutamic-oxaloacetic transaminase, and glutamate pyruvate transaminase, were measured using a dry-chemistry blood analyzer (Spotchem SP-4410, Arklay, Kyoto, Japan), according to the manufacturer’s instructions.

### Assessment of cognitive function

The Barnes maze test was used to evaluate the cognitive function as previously described [3]. Briefly, the Barnes maze test is a standardized behavioral assessment for the evaluation of cognitive development. The maze used in the study was a circular platform with 20 holes at the edge (105 cm height and 92 cm diameter). One of the 20 holes (called escape hole) was linked to a hidden escape cage under the circular platform where the animals were subjected to escape and hide from light distress installed above the platform. Cognitive development was assessed during the final week of the experimental period at the same time each day (between 09:00–13:00) in a dedicated room. Animals were subjected to an adaptation phase (Day 0), a learning phase (Day 1-Day 4), and a test trial (Day 5), during which they learned the experimental environment and task to find the escape cage through the escape hole. During the learning phase, the animals were allowed to freely explore the maze for 3 min to learn where the target hole was located (two training trials per day with a 15 min interval). On the test day, the mice were allowed to find the escape hole in only a single trial. All procedures were video recorded and analyzed using Ethovision XT 10 software (Noldus, Wageningen, Netherlands). Latency (time to reach the escape hole on the test day) was used to measure cognitive function. The relative latency (%) on the test day was calculated by dividing the escape latency (s) achieved on the test day by those achieved on the first day.

### Gut microbial analysis

#### Stool sample collection and genomic DNA extraction

Stool samples were collected and immediately stored at −80°C for later analysis. Representative samples from each group were randomly selected for the gut microbial analysis. According to the manufacturer’s instructions, genomic DNA was isolated from stool samples using a QIAamp Fast DNA Stool Mini Kit (QIAGEN, Hilden, Germany). Extracted genomic DNA was confirmed via gel electrophoresis and quantified by spectrophotometer NanoDrop ND-2000 (Thermo Scientific, Waltham, MA).

#### Amplification of 16S rRNA gene and sequencing

Hypervariable regions (V1-V3) of the 16S ribosomal ribonucleic acid (rRNA) gene were amplified using barcoded universal primers for each sample. The primer sequences used in this study were as follows: V1-9F (5’-CCTATCCCCTG TGTGCCTTGGCAGTC-TCAG-AC-AGAGTTTGATC MTGGCTCAG-3’) and V3-541R (5’-CCATCTCATC CCTGCGTGTCTCCGAC-TCAG-X-AC-ATTACCGC GGCTGCTGG-3’). Polymerase chain reaction (PCR) was performed using BioFact F-Star Taq DNA polymerase (BioFACT, Seoul, Republic of Korea) as previously described [23]. Briefly, a final volume of 50 μL of PCR reaction contained approximately 20 ng of DNA template, 5 μL of 10× Taq buffer (20 mM Mg^2+^), 1 μL of 10 mM dNTP mix, 2 μL of forward and reverse barcoded primers (10 pmol/μL), and 0.25 μL of DNA polymerase. PCR reactions were performed using a GeneAmp PCR system 9700 (Applied Biosystems, Foster City, CA, USA). The PCR program was as follows: initial denaturation for 5 min at 94°C; followed by 28 cycles of denaturation (30 s at 95°C), annealing (30 s at 60°C), and extension (30 s at 72°C) with a final extension step (10 min at 72°C); followed by holding at 4°C. The PCR product was confirmed using 1% agarose gel electrophoresis and visualized using a Gel Doc system (BioRad, Hercules, CA, USA). The amplified products were purified with the PureLink Quick Gel Extraction and PCR Purification Combo Kit (Invitrogen, Carlsbad, CA, USA) and quantified using a Qubit 2.0 fluorometer (Invitrogen). The library size was assessed using a BioAnalyzer (Agilent Technologies, Santa Clara, CA, USA). Amplicons were pooled and sequenced using a 454 GS junior platform (Roche, Germany).

### Bioinformatic analysis of sequencing data

Microbial sequences were processed using the Ribosomal Database Project (RDP) pyrosequencing pipeline (http://pyro.cme.msu.edu/;release11) [24]. Briefly, sequencing reads with (1) one or more ambiguous bases, (2) quality score of less than 20, or (3) less than 300 bp were excluded from the analysis. The RDP Classifier was used for taxonomy-based analysis, and a comparative analysis of the relative abundances of taxa was performed to detect differentially represented taxa across the samples [25]. A comparative analysis of relative abundance between the groups was performed to identify the differential features across the samples.

### Statistical analysis

In the “water restriction experiment,” a comparison analysis of variables between groups was performed using an unpaired t-test or analysis of variance (ANOVA). Selection of a significant gut microbial community was based on fold change ≥ 1.5 (dehydration group vs. control group in juvenile mice) and false discovery rate (FDR) correction on the t-test (p < 0.05). In the “different water source experiment,” a comparison analysis of variables between groups was conducted using ANOVA and Duncan’s test or FDR correction on ANOVA. The relative abundance of the gut microbiota was normalized using the z-score method in the heatmap. The relationship between cognitive development and gut microbial community was evaluated using Spearman’s correlation analysis. P < 0.05 was considered statistically significant. All statistical analyses were conducted using SPSS (version 26.0; SPSS Inc., Chicago, IL, USA) or GraphPad Prism (version 9.3.1; GraphPad Software, San Diego, CA, USA).

## Results

### Insufficient water intake altered gut microbial colonization in infant mice

**Fig 1:**
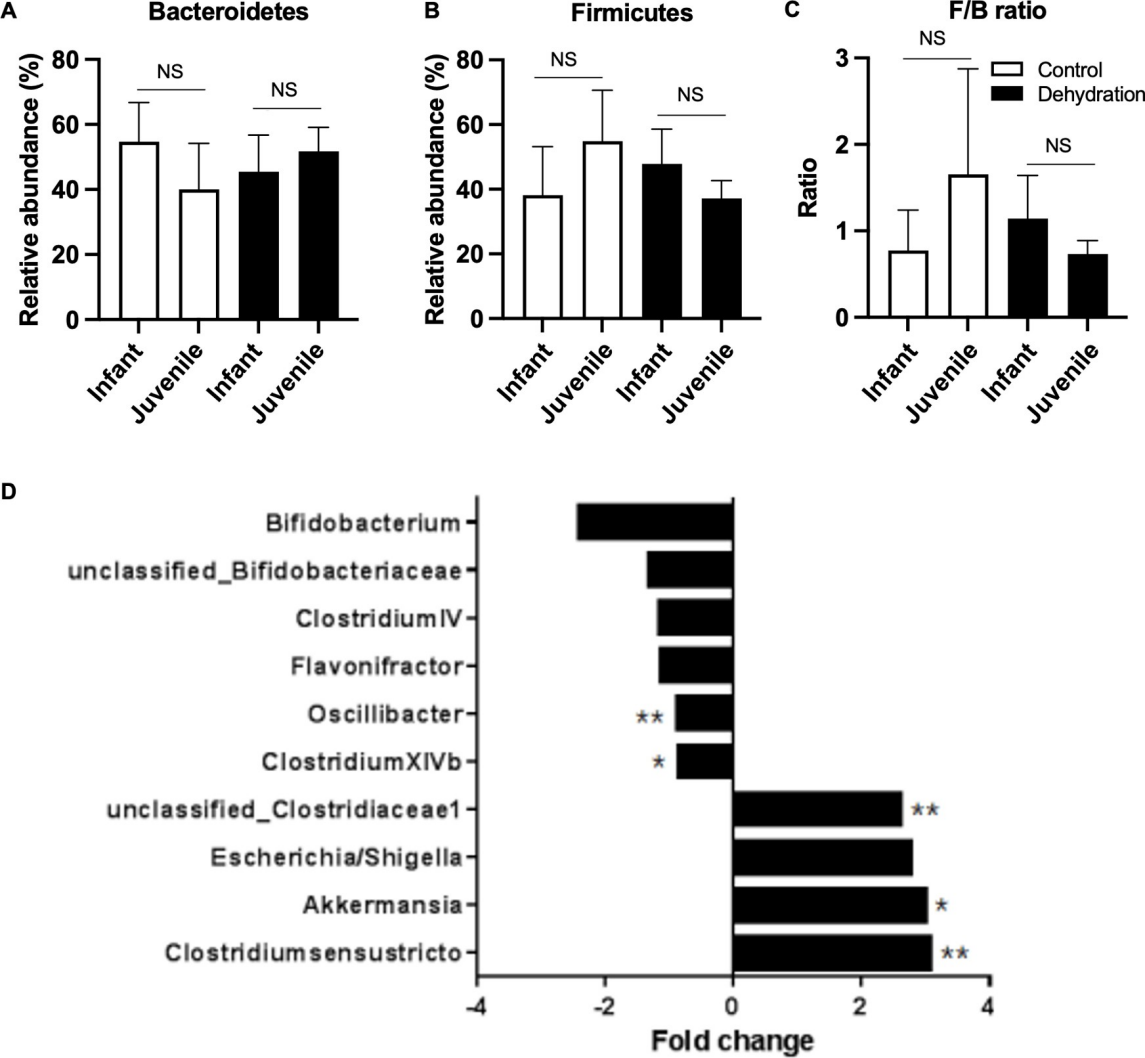
Altered gut microbial composition due to insufficient consumption of drinking water in infant mice. (A-C) Relative abundances of two major phyla, Bacteroidetes and Firmicutes, and Firmicutes-to-Bacteroidetes ratio (F/B ratio) are shown in the control groups (infant and juvenile) and dehydration groups (infant and juvenile). Data are presented as mean ± SD. NS indicates no statistical significance based on FDR correction on ANOVA. (D) Significantly altered genera with a 1.5-fold change in the dehydration group than in the control group in juvenile mice. Statistical significance was based on FDR correction on t-test: *p < 0.1 and **p < 0.05.

A previous study reported that mild dehydration (induced by 60% less drinking water intake) for 4 weeks impaired cognitive development in infant mice [3]. However, the effect of dehydration on gut microbial colonization in infant mice and its association with cognitive development in terms of the gut-brain axis has not been determined. Therefore, we aimed to investigate the effects of dehydration on the normal development of gut microbiota in infant mice and its association with cognitive development. First, we compared the gut microbial communities between infant (4-week-old) and juvenile mice (7-week-old) with sufficient drinking water intake. We found that intestinal bacterial phyla were significantly different between infant and juvenile mice, with Bacteroidetes showing a decreasing tendency in juvenile mice than in infant mice (40.07% vs. 54.67%, **Fig 1A**). The relative abundance of Firmicutes showed a different tendency as it was increased in juvenile mice than in infant mice (54.89% vs. 38.09%) (**Fig 1B**), resulting in an increased tendency in Firmicutes-to-Bacteroidetes ratio (F/B ratio) in juvenile mice than in infant mice (1.66 vs. 0.77). These results indicated that the gut microbiota in infant mice was still developing (**Fig 1C**). Next, we examined whether insufficient drinking water intake influenced the normal colonization of the gut microbiota. We compared the relative abundance of the gut microbial composition at various taxonomic levels in infant and juvenile mice with mild dehydration. Notably, dehydration reversed the bacterial changes observed during the normal developmental period. Although statistically insignificant, the relative abundances of Bacteroidetes and Firmicutes in dehydrated juvenile mice (7-week-old, Bacteroidetes, 37.15%; Firmicutes, 51.73%) were reduced to levels similar to those of normal infant mice (4-week-old, Bacteroidetes, 38.09%; Firmicutes, 54.67%; **Fig 1A and 1B**). These shifts in the two major phyla resulted in changes in the F/B ratio, showing that the increasing tendency of the F/B ratio during the developmental period was reversed by insufficient water intake, reducing the F/B ratio in dehydrated juvenile mice to that seen in normal infant mice (dehydrated juvenile mice [0.73] vs. normal infant mice [0.77]) (**Fig 1C**). Next, we evaluated whether dehydration induced alterations in gut flora at the genus level. We identified 10 genera differentially distributed by dehydration, with a 1.5-fold change in juvenile mice, including *Bifidobacterium*, unclassified Bifidobacteriaceae, *Clostridium IV*, *Flavonifractor*, *Oscillibacter*, *Clostridium XIVb*, unclassified_Clostridiaceae1, *Escherichia/Shigella*, *Akkermansia*, and *Clostridium sensu stricto* (**Fig 1D**). Among these 10 genera, we found three genera with statistical significance, including *Oscillibacter*, unclassified_Clostridiaceae1, and *Clostridium sensu stricto* (p < 0.05, **Fig 1D**), showing that insufficient water intake during the developmental period altered the normal colonization of the intestinal bacterial community.

### Drinking water source did not significantly affect gut microbial composition

**Fig 2:**
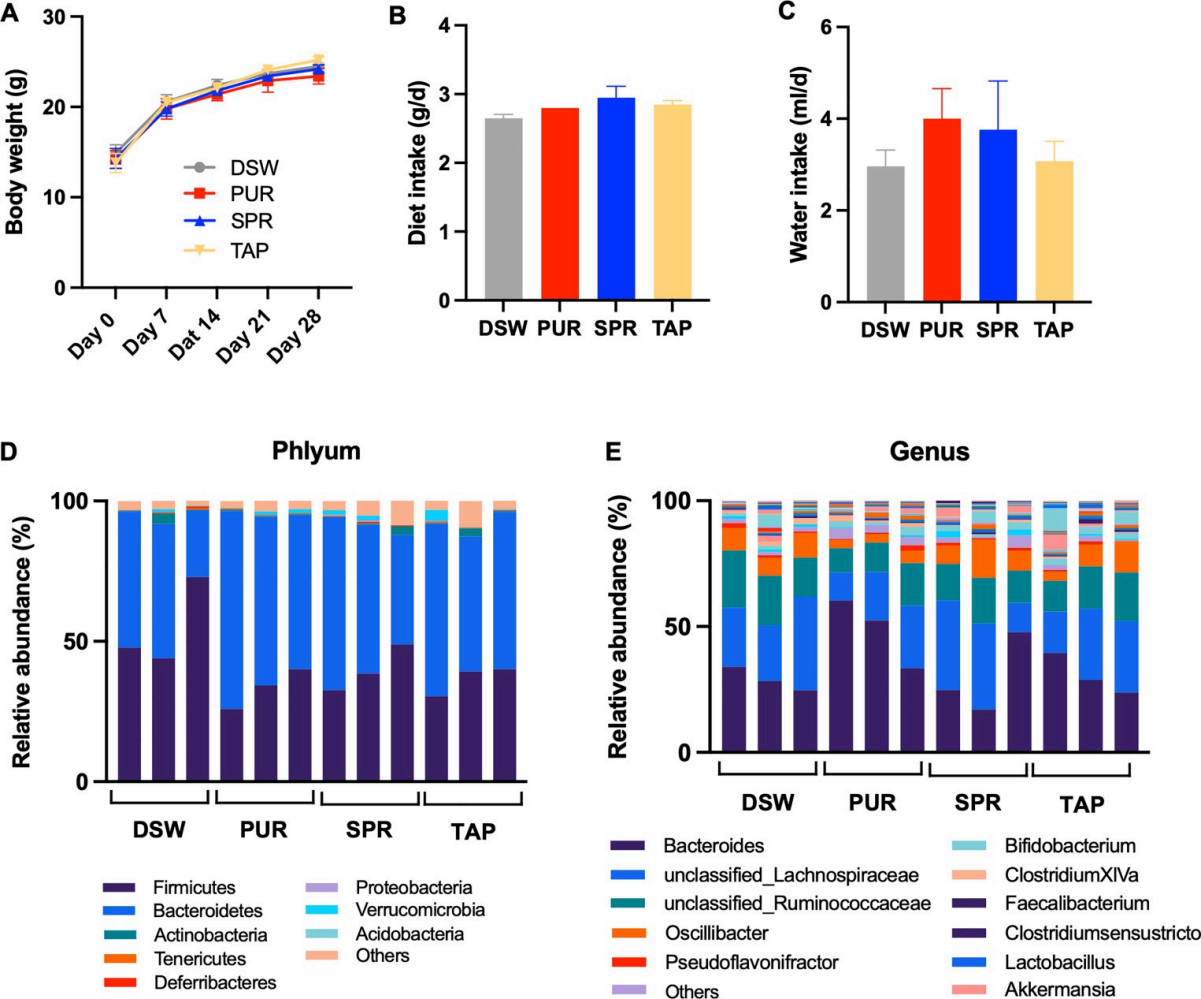
Physical and physiological status and gut microbial composition depending on the sources of drinking water. (A-C) Body weight and food and water intake were measured throughout the experimental period in the different water source groups. Data are presented as mean ± SD. (D-E) Relative abundances of gut microbiota at phylum and genus levels are shown as bar charts in the different water source groups. Abbreviations: Distilled water (DSW), purified water (PUR), spring water (SPR), and tap water (TAP) groups. Statistical significance was based on ANOVA and Duncan’s test or FDR correction on ANOVA: *p < 0.1 and **p < 0.05.

To understand the effect of consuming drinking water from various sources on intestinal flora, animals were provided with different drinking water sources, including DSW, PUR, SPR, and TAP, for 4 weeks. The water components from the different water sources are summarized in **S1 Table**. As expected, the mineral content differed across the water source groups, showing that the total dissolved solids were higher in SPR, TAP, PUR, and DSW in that order (**S1 Table**). In addition, the pH was similar in all water sources except for the DSW, which had a lower pH (pH 4.9). Therefore, we first evaluated whether the different concentrations of mineral contents and drinking water pH affected the physical and physiological status by measuring body weight and biochemical markers in the blood. We confirmed that the dietary and drinking water intake did not change depending on the source of drinking water (**Fig 2A–2C**) and blood biochemical indices, including serum glucose, total cholesterol, blood urea nitrogen, and liver function indices (total bilirubin, glutamic-oxaloacetic transaminase, and glutamate pyruvate transaminase) were not significantly different between the groups (**Table 1**).

**Table 1:** Blood biochemical characteristics across different water source groups.

Characteristics	DSW	PUR	SPR	TAP	P-value
**Glucose (mg/dL)**	248 ± 30	230 ± 19	215 ± 30	239 ± 26	0.21
**Total cholesterol (g/dL)**	107 ± 17	94 ± 10	87 ± 23	93 ± 7	0.18
**Blood urea nitrogen (mg/dL)**	29 ± 2	32 ± 3	27 ± 2	26 ± 3	0.15
**Total bilirubin (mg/dL)**	0.42 ± 0.30	0.50 ± 0.32	0.32 ± 0.15	0.25 ± 0.08	0.29
**Glutamic-oxaloacetic transaminase**	93 ± 32	77 ± 30	70 ± 21	78 ± 21	0.92
**Glutamate pyruvate transaminase**	29 ± 17	21 ± 14	18 ± 10	19 ± 5	0.49

Data are presented as mean ± SD. Abbreviations: Distilled water (DSW), purified water (PUR,), spring water (SPR), and tap water (TAP) groups. The P-value was from ANOVA.

**Fig 3:**
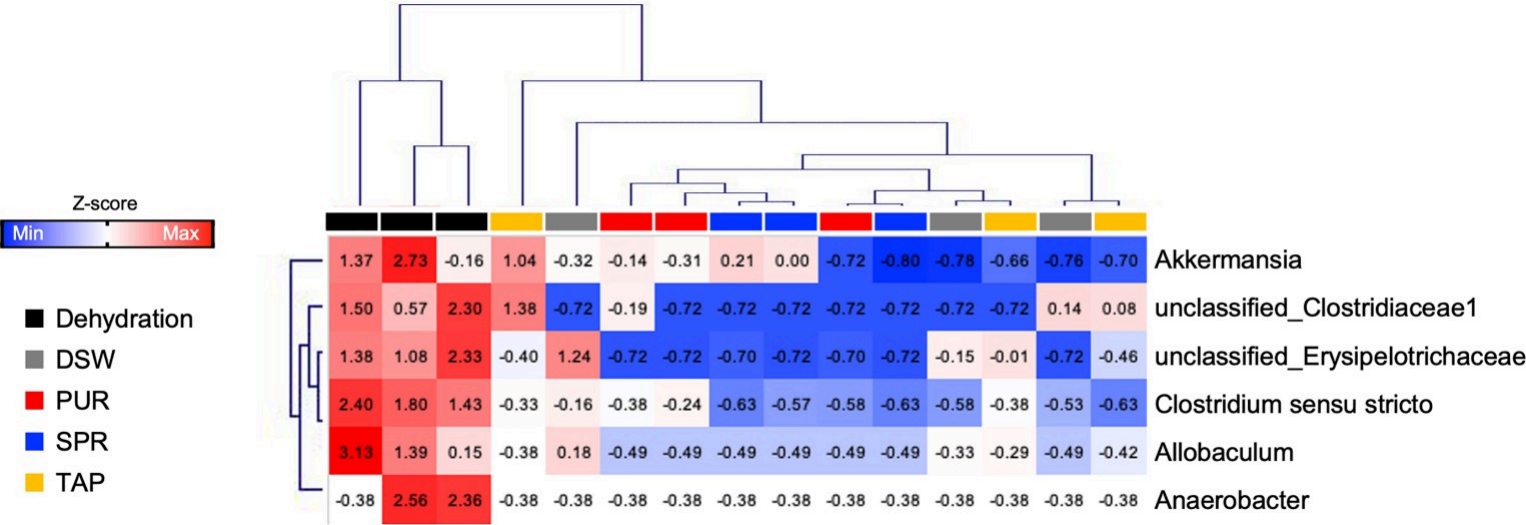
Gut microbial composition depending on the amount of drinking water consumed. Hierarchical clustering analysis showing the six genera that contributed to the clear distinction between mice with limited access to drinking water and mice from the other groups that consumed different types but sufficient amounts of water (DSW, PUR, SPR, and TAP). Values in the heatmap indicate the z-scores of relative abundances of six genera that were significantly different based on the t-test (p < 0.01). Red and blue colors in the color key indicate high and low abundances, respectively. Each column presents each individual mouse. Abbreviations: Distilled water (DSW), purified water (PUR), spring water (SPR), and tap water (TAP) groups.

Next, to understand the effect of the different mineral content of drinking water on the gut microbiota, we compared the gut bacterial composition of mice from the different water source groups. First, we identified that the composition of the intestinal flora at the phylum (**Fig 2D**) and genus levels (**Fig 2E**) were not significantly different from the other water source groups. Additionally, we conducted a clustering analysis to identify which factor had a more determinant role in shaping the gut microbial composition between the water quantity intake and types of drinking water. Notably, fecal samples were distinguished based on hierarchical clustering analysis, showing that fecal samples in the dehydration group were clearly clustered from the fecal samples from groups that consumed different types but sufficient amounts of water (DSW, PUR, SPR, and TAP) (**Fig 3**). In addition, we found that six genera, including *Akkermansia*, unclassified*_*Clostridiaceae1, unclassified_Erysipelotrichaceae, *Clostridium sensu stricto*, *Allobaculum*, and *Anaerobacter*, constituted bacteria that contributed to the distinct difference between mice with limited access to drinking water and those that consumed water *ad libitum* (p < 0.01, **Fig 3**). These results suggested that the type of drinking water did not affect the gut flora; rather, the water quantity intake was an imperative factor affecting the intestinal microbiota.

### Cognitive function was associated with gut microbial profiles

**Fig 4:**
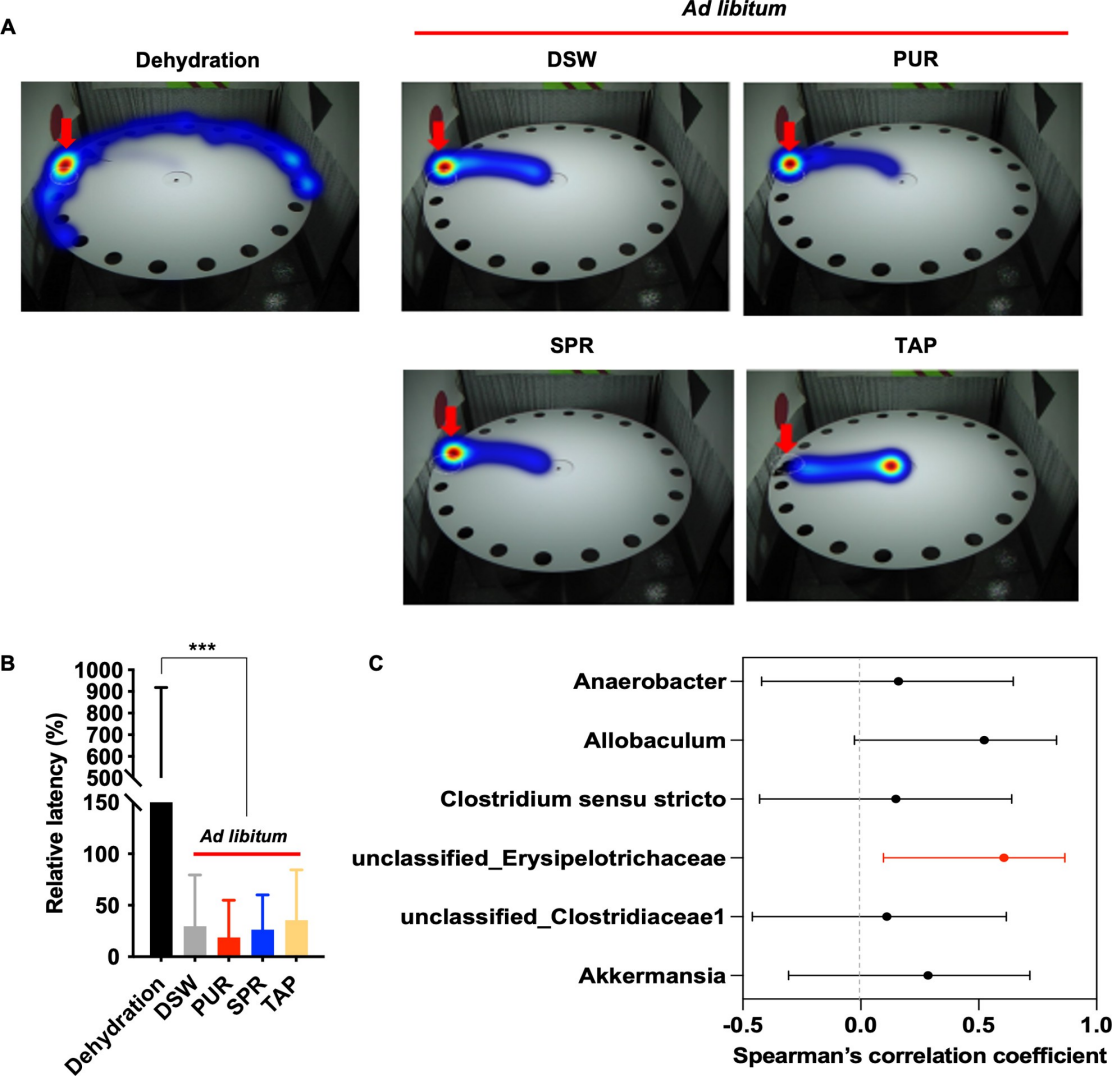
Relationship between cognitive development and gut microbial community depending on the amount of drinking water consumed. (A) Representative heatmap images of the tracking path through the maze of mice in each group on the final test day of the Barnes maze test. Heatmap and arrow indicate time spent at each position and target escape hole, respectively. (B) Relative latency (%) is calculated by the escape latency (s) achieved on the test day divided by that achieved on the first day. Data are presented as mean ± SD. Statistical significance was based on a t-test: ***P <0.005. (C) Spearman’s correlation coefficients with 95% confidence intervals are shown as a forest plot. Significant correlation (p < 0.05) is marked in red color. Abbreviations: Distilled water (DSW), purified water (PUR), spring water (SPR), and tap water (TAP) groups.

In a previous study, we confirmed that drinking water consumption had an important effect on cognitive development in infant mice [3]. To further examine whether the type of drinking water and its amount had a different effect on the regulation of the gut-brain axis and cognitive development, cognitive development was measured in the different water source groups using the Barnes maze test. In the last week of the experiment, the time to find the target hole during the 4-day learning period and the test day was measured. As previously described, the dehydrated mice showed impaired cognitive function with behavioral alterations in locating the target hole on the last day of the Barnes maze test. **Fig 4A** shows the representative heatmap images of the tracking path through the maze by mice in each group on the final test day of the Barnes maze test. The mice that consumed sufficient drinking water in the DSW group directly headed to the target escape hole (**Fig 4A**). In addition, there were no significant differences in the relative latency between the groups with different sources of drinking water (**Fig 4A and 4B**); however, there was a significant difference depending on the amount of drinking water intake between the dehydration group versus the groups that consumed sufficient water. The relative latency on the test day was significantly higher in the dehydration group (391.32 ± 527.49%) than in the groups that consumed sufficient water (DSW: 29.72 ± 49.83%, PUR: 18.72 ± 36.21%, SPR: 26.34 ± 33.84%, and TAP: 35.47 ± 48.97%) (p < 0.005, **Fig 4B**). Therefore, these results indicated that the source of drinking water during infancy did not have a significant effect on cognitive development. We further examined whether alterations in the gut microbial composition were associated with cognitive decline in the dehydration group. We conducted a correlation analysis between the relative latency and relative abundance of bacterial communities that changed due to insufficient water consumption. Interestingly, we found a statistically significant positive association between unclassified_Erysipelotrichaceae and relative latency (r = 0.61; 95% confidence interval of r: 0.10–0.87; p < 0.05, **Fig 4C**), indicating that relative latency increased with the increase in unclassified_Erysipelotrichaceae.

## Discussion

In this study, we aimed to determine whether the quantity and type of drinking water affect physiological and biological functions, including brain function, by confirming how it affects gut microbiota which has an important regulatory role in host physiology. We found that an insufficient intake rather than the source of drinking water was a contributing factor for shaping normal gut microbial composition in infant mice, and microbial changes were closely linked to altered cognitive function in mice with dehydration.

The gut microbiota community undergoes significant changes with age [26]. A study reported that the F/B ratio increased from early life to adulthood [26], which is consistent with our finding that showed an increased F/B ratio during the developmental period from infancy to juvenile. Notably, this shift in the F/B ratio was reversed by dehydration, which indicates that proper consumption of drinking water contributes to the normal development of intestinal flora.

The infant gut microbiota is implicated in the normal development and maturation of neurological functions [27–29]. Therefore, inadequate colonization during the early days of life may increase susceptibility to neuronal disorders [11]. Indeed, the increased abundance of Erysipelotrichaceae, as found in our study, has been suggested to be a potential contributor to the gut-brain axis [30]. Neonatal antibiotic administration in mice causes gut dysbiosis, with a large increase in Erysipelotrichaceae and disrupted hippocampal neurogenesis, leading to cognitive impairment in adulthood [30]. In addition, neuropsychological behaviors induced by chronic alcohol exposure in mice were associated with gut microbial dysbiosis, with an elevation in the abundance of *Allobaculum spp*. [31]. In this study, *Allobaculum spp*. also showed a negative correlation with changes in brain-derived neurotrophic factor in the prefrontal cortex and hippocampus [31]. These results support our findings that elevated Erysipelotrichaceae and *Allobaculum spp*. levels in dehydrated mice may play a vital role in brain function. Several pathways within the gut-brain axis account for the microbial influence on brain development, including pathways involving the vagus nerve, metabolites, and immune system [27]. Indeed, gut microbial maturation at weaning has been recognized as a regulator of the development of the immune system, which is one of the key factors in the gut-brain axis [32]. Therefore, our results may suggest that disruption of the gut microbial community during development by insufficient water intake increases the risk of inflammatory disorders with abnormal brain function and behavior [27].

Aside from gut microbiota’s role in cognitive function, gut dysbiosis in infants is also recognized for its association with various immune and metabolic disorders such as asthma, atopic dermatitis, inflammatory bowel disease, and type 1 diabetes [28]. Interestingly, our findings indicated that insufficient drinking water consumption during infancy caused changes in the gut microbial communities, including several genera related to immune and metabolic disorders. An increase in *Clostridium sensu stricto* has been positively associated with atopic dermatitis [33] and food allergy [34] in early childhood. In addition, an increased abundance of *Akkermansia* has been associated with eczema [35] and atopic dermatitis in infants [36]. Additionally, *Oscillibacter* produces valeric acid, which is involved in the differentiation of interleukin 10 (IL-10)-producing Tregs [37, 38], supporting our findings that a reduced abundance of *Oscillibacter* in dehydrated mice may result in immature immune function in infants. Moreover, the increased abundance of the family Erysipelotrichaceae in dehydrated mice is relevant because Erysipelotrichaceae has been associated with atopic dermatitis in infants [39]. Studies have also demonstrated that the expansion of Erysipelotrichaceae, specifically *Allobaculum spp*., (which corroborates our findings) may be responsible for the development of obesity via dysregulation of cholesterol metabolism [40, 41]. Collectively, an imbalance in these genera due to insufficient consumption of drinking water during early life affects infant health, which may cause a number of long-term health challenges, such as allergic and metabolic diseases and the development of cognitive function.

The biological effects of the mineral composition of drinking water have been an issue. Several studies have argued that calcium present in drinking water may be a significant source of calcium, which may influence calcium homeostasis [42]. In addition, case-control studies reported that the amount of magnesium in drinking water was inversely associated with the risk of death due to heart infarction, but a causal relationship between the supply of magnesium from drinking water and the risk of disease has not yet been proven [43, 44]. In particular, the effects of different mineral concentrations in drinking water on the gut microbial community have not been explored. Although a recent study reported the association of the drinking water source with gut microbial composition in a population-based cohort [45], key limitations existed. They reported that the types of drinking water affected the fecal microbiota, indicating that individuals drinking well water had a reduced abundance of the genera *Odoribacter*, *Bacteroides*, and *Streptococcus* than those drinking bottled and city water [45]. However, the study results relied on self-reported and subjective measures, and it was difficult to control the various confounding factors significantly affecting the gut microbiota. To our knowledge, the present well-controlled animal study is the first to show that drinking water with different mineral content does not significantly affect physical and physiological changes in gut microbial composition. Moreover, the pH of DSW was lower (pH 4.9) than that of the other water sources that had neutral pH. Thus, the difference in pH of drinking water also did not significantly affect the intestinal microbial flora in our study, although an early study showed that extremely acidic (pH 3.0–3.2) drinking water altered the diversity and composition of the gut microbiota in mice [46].

The present study was limited in that although it explored the gut microbial composition at various taxonomic levels, further analyses of the functional characteristics of the gut microbiome using metagenomic sequencing and metabolome profiles are warranted to better understand the interaction between the host and microbiome. Nonetheless, the well-controlled design of the present study enabled us to understand the health impacts related to the amount of drinking water consumed and the mineral intake from drinking water, focusing on the gut microbial composition.

## Conclusion

This is the first study suggesting that mineral content in drinking water does not matter; rather, the water quantity intake is an imperative factor for shaping the early gut microbiota associated with cognitive development during infancy.

## Supporting information

S1 Fig Experimental design. Three-week-old mice were subjected to 1) a water restriction experiment or 2) a different water source experiment. In the “water restriction experiment,” animals were randomly assigned to one of the following experimental groups: control groups (infant mice group, n = 5; juvenile mice group, n = 6); dehydration groups (infant mice group, n = 6; juvenile mice group, n = 8). Mice in the dehydration groups had limited access to water bottles (15 min/d) to induce mild dehydration, whereas animals in the control groups consumed water *ad libitum*. In the “different water source experiment,” animals were randomly assigned to the following experimental groups: distilled water group (DSW; n = 6), purified water group (PUR; n = 6), spring water group (SPR; n = 6), and tap water group (TAP; n = 6). Animals in the DSW, PUR, SPR, and TAP groups consumed water *ad libitum*.
(PDF)

S1 Table Water composition of the different water sources. Abbreviations: Distilled water (DSW), purified water (PUR), spring water (SPR), and tap water (TAP) groups.
(DOCX)
