# Supplementary material for: Gut microbiota and cognitive development in infant mice: Quantity and source of potable water
Source: PLoS One. 2023 Jun 14;18(6):e0286951. doi: 10.1371/journal.pone.0286951 (PMC10266684; doi:10.1371/journal.pone.0286951)
Supplement: S1 Table — Abbreviations: Distilled water (DSW), purified water (PUR), spring water (SPR), and tap water (TAP) groups. (DOCX) [file pone.0286951.s002.docx]

**S1 Table. Water composition of the different water sources**

| **Characteristics** | **DSW** | **PUR** | **SPR** | **TAP** |
| --- | --- | --- | --- | --- |
| **pH** | 4.9 | 6.8 | 7.7 | 7.5 |
| **Total Dissolved Solids (TDS, mg/L)** | 1.9 | 18.0 | 267.2 | 100.9 |
| **Copper (Cu, mg/L)** | <0.001 | 0.041 | <0.001 | 0.509 |
| **Silicon (Si, mg/L)** | <0.001 | <0.001 | 6.850 | 0.420 |
| **Sodium (Na, mg/L)** | <0.001 | 1.950 | 5.970 | 7.420 |
| **Magnesium (Mg, mg/L)** | <0.001 | <0.001 | 28.270 | 3.950 |
| **Barium (Ba, mg/L)** | <0.001 | <0.001 | 0.099 | 0.014 |
| **Strontium (Sr, mg/L)** | <0.001 | 0.011 | 0.344 | 0.091 |
| **Zinc (Zn, mg/L)** | <0.001 | 0.007 | <0.001 | 0.078 |
| **Silica (SiO_2,_ mg/L)** | <0.001 | <0.001 | 14.800 | 0.910 |
| **Potassium (K, mg/L)** | <0.001 | 0.430 | 1.010 | 2.060 |
| **Calcium (Ca, mg/L)** | <0.001 | 2.720 | 79.380 | 22.580 |
| **Chromium (Cr, mg/L)** | <0.001 | <0.001 | <0.001 | <0.001 |
| **Lead (Pb, mg/L)** | <0.001 | <0.001 | <0.001 | <0.001 |
| **Nickel (Ni, mg/L)** | <0.001 | <0.001 | <0.001 | <0.001 |
| **Boron (B, mg/L)** | <0.001 | <0.001 | <0.001 | <0.001 |
| **Arsenic (As, mg/L)** | <0.001 | <0.001 | <0.001 | <0.001 |
| **Selenium (Se, mg/L)** | <0.001 | <0.001 | <0.001 | <0.001 |
| **Aluminum (Al, mg/L)** | <0.001 | <0.001 | <0.001 | <0.001 |
| **Silver (Ag, mg/L)** | <0.001 | <0.001 | <0.001 | <0.001 |
| **Phosphorus (P, mg/L)** | <0.001 | <0.001 | <0.001 | <0.001 |
| **Iron (Fe, mg/L)** | <0.001 | <0.001 | <0.001 | <0.001 |
| **Cadmium (Cd, mg/L)** | <0.001 | <0.001 | <0.001 | <0.001 |
| **Manganese (Mn, mg/L)** | <0.001 | <0.001 | <0.001 | <0.001 |

Abbreviations: Distilled water (DSW), purified water (PUR), spring water (SPR), and tap water (TAP) groups.
